# Supplementary material for: Twenty-Year Survival of Patients Operated on for Non-Small-Cell Lung Cancer: The Impact of Tumor Stage and Patient-Related Parameters
Source: Cancers (Basel). 2022 Feb 10;14(4):874. doi: 10.3390/cancers14040874 (PMC8870355; doi:10.3390/cancers14040874)
Supplement: Supplementary file 1 [file cancers-14-00874-s001.zip › cancers-1581911-supplementary.pdf]

cancers

MDPI

Supplementary Materials

Table S1. Multivariate analysis of factors influencing risk of death at 20 years by Cox proportional hazard models after adjustment on CRP. The Model 3 correspond to the Model 1 adjusted to pre-operative CRP. The Model 4 correspond to Model 2 adjust to CRP.

Variable	Relative Risk (RR)	95% CI	*p*
**Model 3 correspond to model 1 adjusted to CRP**			
p=0.000058 model			
Number of patients at multivariate n=270			
Age			
< 50 yrs	1		
50 yrs - 60 yrs	1.21	(1.06-1.38)	
60 yrs - 70 yrs	1.46	(1.11-1.91)	
70 yrs ≤	1.76	(1.18-2.64)	0.006
**CRP (mg/ml)**			
CRP ≤ 3 mg/ml	1		
3 mg/ml < CRP	1.33	(1.01-1.75)	**0.041**
**Pathological stages (7 classes)**			
**IA**	1		
**IB**	1.08	(1.00-1.17)	
**IIA**	1.18	(1.00-1.38)	
**IIB**	1.28	(1.01-1.62)	
**IIIA**	1.38	(1.01-1.90)	
**IIIB**	1.50	(1.01-2.23)	
**IV**	1.63	(1.01-2.62)	**0.045**
Cumulative smoking			
< 20 p./yr	1		
20 p./yr-50 p./yr	1.22	(1.00-1.49)	
50 p./yr-100 p./yr	1.49	(1.00-2.21)	
100 p./yr ≤	1.82	(1.00-3.28)	0.048
BMI kg/m			
BMI < 18.5	1		
18.5 ≤ BMI < 25	0.81	(0.66-0.99)	
25 ≤ BMI	0.65	(0.43-0.99)	0.044
Chronic bronchitis			
Not	1		
Yes	1.34	(0.97-1.84)	0.076
COPD Yes/not			0.28
Sex			0.28
COPD GOLD			0.59
Diabetes mellitus			
Yes/not			0.40
Weight loss			
<5% 5%-10% 10%≤			0.53
Type of resection			

Pneumonec./ bilob. or lob.			0.69
Histological type			
Aden./Squa./Lar.cell/ others			0.87
Past or current smoker			
Yes/not			0.85
Alcohol abuse			
Yes/not			0.89
**Variable**	**Relative Risk (RR)**	**95% CI**	***p***
p=0.00000018 model			
Number of patients at multivariate n= 270			
**Model 4 correspond model 1 adjusted to CRP and FEV1**			
**FEV1 preoperative (%)**			
**<50%**	1		
**50% - 60%**	0.75	(0.66-0.86)	
**60% - 70%**	0.57	(0.44-0.74)	
**70% -_ 80%**	0.43	(0.29-0.63)	
**80% ≤**	0.32	(0.19-0.54)	**0.000018**
COPD			
GOLD1	1		
GOLD2	0.63	(0.47-0.85)	
GOLD3	0.39	(0.22-0.72)	0.0025
Chronic bronchitis			
not	1		
**yes**	1.49	(1.09-2.03)	0.012
Age (years)			
< 50 yrs	1		
50 yrs - 60 yrs	1.20	(1.05-1.37)	
60 yrs - 70 yrs	1.44	(1.10-1.88)	
70 yrs ≤	1.73	(1.16-2.58)	0.0075
**Pathological stages (7 classes)**			
**IA**	1		
**IB**	1.10	(1.02-1.20)	
**IIA**	1.21	(1.03-1.43)	
**IIB**	1.34	(1.05-1.71)	
**IIIA**	1.48	(1.07-2.04)	
**IIIB**	1.63	(1.08-2.44)	
**IV**	1.79	(1.10-2.92)	0.019
BMI kg/m2			
BMI < 18.5	1		
18.5 ≤ BMI < 25	0.77	(0.63-0.95)	
25 ≤ BMI	0.60	(0.39-0.90)	0.014
**CRP (mg/ml)**			
**CRP ≤ 3 mg/ml**	1		
**3 mg/ml < CRP**	1.35	(1.02-1.78)	**0.037**
Cumulative smoking			
< 20 p./yr	1		
20 p./yr _ 50 p./yr	1.21	(1.00-1.48)	
50 p./yr - 100 p./yr	1.47	(0.99-2.19)	
100 p./yr ≤	1.79	(0.99-3.23)	0.053
Weight loss			
<5% 5%-10% 10%≤			0.33
Sex			0.42
Diabetes mellitus			
Yes/not			0.39
Histological type			
Aden./Squa./Lar.cell/ others			0.48
Type of resection			
Pneumonec./ bilob. or lob.			0.72
COPD Yes/not			0.85
Alcohol abuse			

Yes/not	0.84
Past or current smoker	
Yes/not	0.98

The relative risks are detailed only for risk factor with a p value ≤0.1 at multivariate by Cox models. + These including sarcomatoid carcinomas and adenosquamous carcinomas. IIIC patients with N3 diseases are not never operated. GOLD4 patient with preoperative VEMS below 30% are not operable.

Suplemental Figure S1

B2

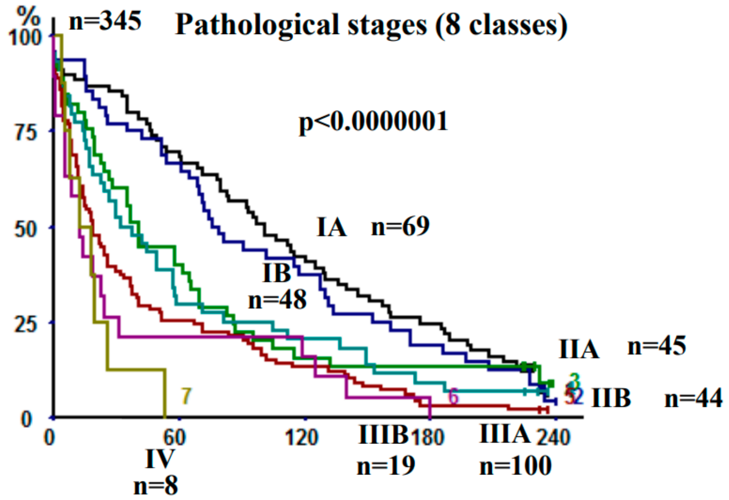

Figure S1

E2

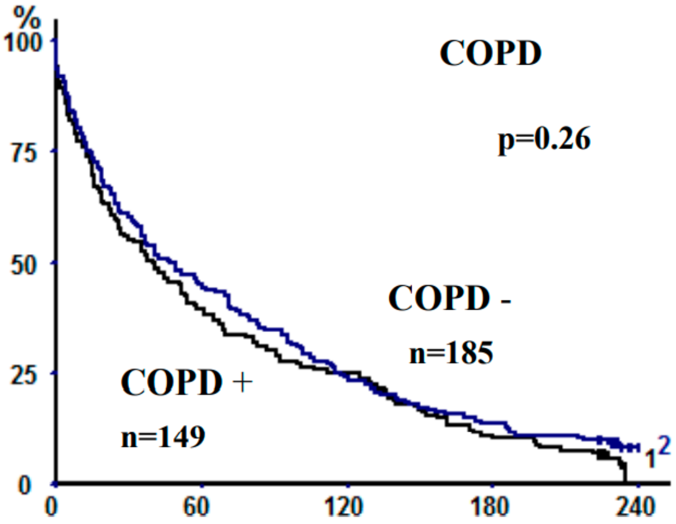

Figure S1

E3

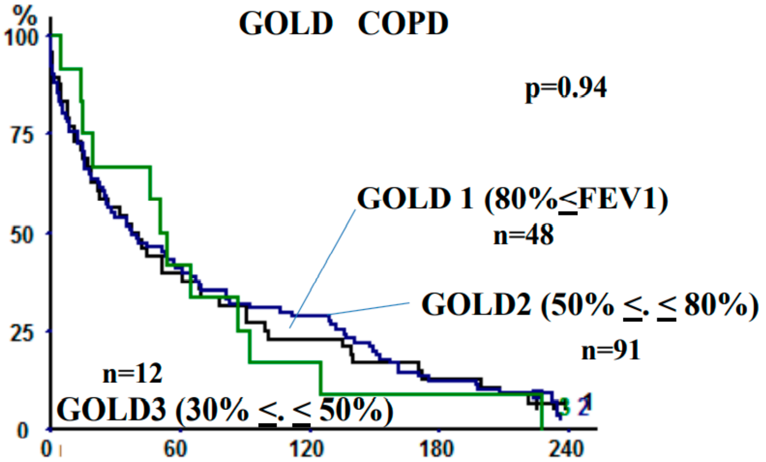

Figure S1

E4

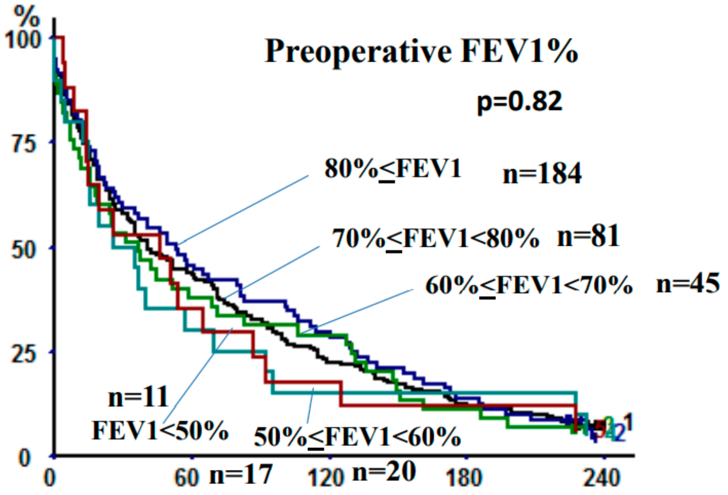

Figure S1

E5

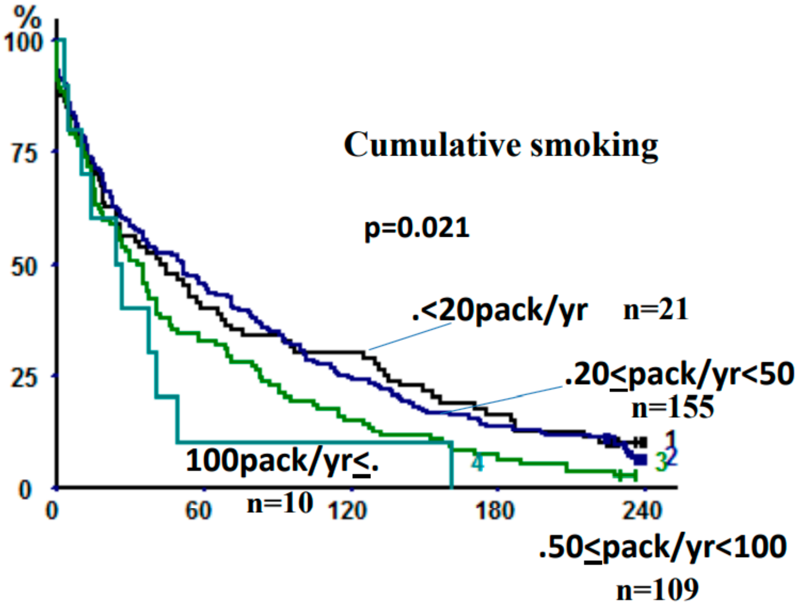

Figure S1

E6

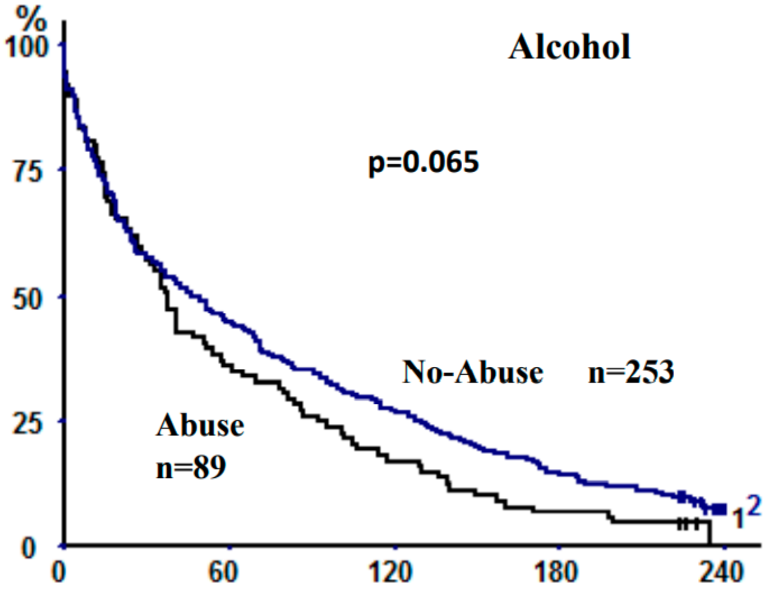

Figure S1

E7

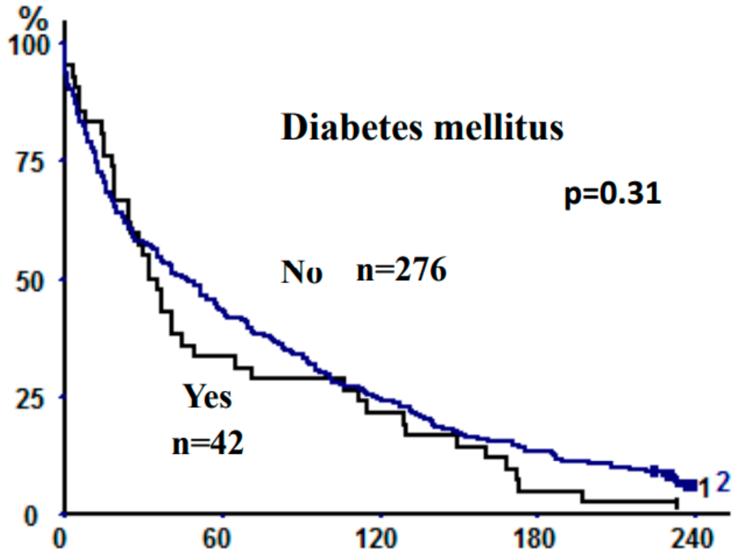

Figure S1. Pathological stages (8 classes) (panel B2), COPD (panel E2), GOLD COPD (panel E3), preopera-tive FEV1% (panel E4), cumulative smoking (panel E5), alcohol (panel E6), diabetes melli-tus (pan-el E7).
